# Phytosulfokine α (PSKα) delays senescence and reinforces SUMO1/SUMO E3 ligase SIZ1 signaling pathway in cut rose flowers (*Rosa hybrida* cv. Angelina)

**DOI:** 10.1038/s41598-021-02712-2

**Published:** 2021-12-01

**Authors:** Morteza Soleimani Aghdam, Amin Ebrahimi, Morteza Sheikh-Assadi

**Affiliations:** 1 Department of Horticultural Science, Imam Khomeini International University, 34148-96818 Qazvin, Iran; 2 Department of Agriculture and Plant Breeding, Faculty of Agriculture, Shahrood University of Technology, Semnan, Iran; 3 Department of Horticultural Science, University College of Agriculture and Natural Resources, University of Tehran, Karaj, Iran

**Keywords:** Plant sciences, Plant hormones, Plant physiology, Plant signalling, Plant stress responses

## Abstract

Roses are widely used as cut flowers worldwide. Petal senescence confines the decorative quality of cut rose flowers, an impressively considerable economic loss. Herein, we investigated the SUMO1/SUMO E3 ligase SIZ1 signaling pathway during bud opening, and petal senescence of cut rose flowers. Our results exhibited that the higher expression of *SUMO1* and *SUMO E3 ligase SIZ1* during bud opening was accompanied by lower endogenous H_2_O_2_ accumulation arising from higher expression and activities of *SOD*, *CAT*, *APX*, and *GR*, promoting proline accumulation by increasing *P5CS* expression and activity and enhancing GABA accumulation by increasing *GAD* expression and activity. In harvested flowers, lower expressions of *SUMO1* and *SUMO E3 ligase SIZ1* during petal senescence were associated with higher endogenous H_2_O_2_ accumulation due to lower expression and activities of *SOD*, *CAT*, *APX*, and *GR*. Therefore, promoting the activity of the GABA shunt pathway as realized by higher expression and activities of *GABA-T* and *SSADH* accompanied by increasing *OAT* expression and activity for sufficiently supply proline in rose flowers during petal senescence might serve as an endogenous antisenescence mechanism for slowing down petals senescence by avoiding endogenous H_2_O_2_ accumulation. Following phytosulfokine α (PSKα) application, postponing petal senescence in cut rose flowers could be ascribed to higher expression of *SUMO1* and *SUMO E3 ligase SIZ1* accompanied by higher expression and activities of *SOD*, *CAT*, *APX*, and *GR*, higher activity of GABA shunt pathway as realized by higher expression and activities of *GAD*, *GABA-T*, and *SSADH*, higher expression and activities of *P5CS* and *OAT* for supplying proline and higher expression of *HSP70* and *HSP90*. Therefore, our results highlight the potential of the PSKα as a promising antisenescence signaling peptide in the floriculture industry for postponing senescence and extending the vase life of cut rose flowers.

## Introduction

Rose flowers (*Rosa hybrida*) are extensively used as cut flowers worldwide. Petal senescence confines the decorative quality of cut rose flowers, an impressively considerable economic loss. Therefore, revealing mechanisms regulating rose flowers' bud opening and petal senescence would be advantageous for introducing an innovative approach for extending the vase life of cut rose flowers^1^.

In plants, SUMO E3 ligase SIZ1 is accountable for the reversible attachment of small ubiquitin-like modifier (SUMO) peptides to target proteins. As a reversible post-translational modification (PTM), SUMOylation regulates plant development and stress responses by holding protein stability, enzymatic activity, subcellular location, and protein–protein interactions^2^. The following are required for SUMOylation: (1) maturation of SUMO peptide by SUMO endopeptidase; (2) SUMO activation by hydrolyzing ATP via E1 SUMO activation enzymes’ (3) SUMO conjugation by E2 SUMO conjugation enzymes; and (4) ligation by E3 SUMO ligase SIZ1 for the attachment of SUMO peptide to a lysine residue of target proteins. In addition, the SUMO isopeptidase enzyme is accountable for deSUMOylation by releasing and recycling SUMO from proteins. Moreover, following the activity of SUMO E4 ligase, polySUMOylation is responsible for the ubiquitination of proteins, which are degraded by ubiquitin E4 ligases. E3 SUMO ligase SIZ1 promotes the activity of E3 ubiquitin ligase COP1 by SUMOylation, whereas E3 ubiquitin ligase COP1 suppresses it by ubiquitination^2,3^. During senescence and stresses, inducer of CBF expression 1/C-repeat binding factors (ICE1/CBFs) signaling pathway^4,5^, heat shock transcription factors/heat shock proteins (HSFs/HSPs) signaling pathway^6,7^, sugar sensing, and signaling by the target of rapamycin/sucrose non-fermenting 1 (SNF1)-related kinase 1 (TOR/SnRK1) signaling pathway^8^, and nitrate reductase/R2R3-MYB transcription factors (NR/MYBs) signaling pathway^9–14^ by SUMO E3 ligase SIZ1 might be accountable for regulating plant growth and development and stress responses.

As a plant peptide hormone, phytosulfokine α (PSKα; Tyr(SO_3_H)-Ile-Tyr(SO_3_H)-Thr-Gln) has been employed to palliate chilling injury and fungal decay, delay senescence, and preserve sensory and nutritional quality in fruits and vegetables during postharvest life. Following the application of PSKα, the activation of a cytosolic cGMP signaling pathway^15,16^ was accompanied by the promotion of the extracellular ATP signaling pathway^17^, increasing the activities of intracellular SnRK1 and SUMO E3 ligase SIZ1 signaling pathways^18,19^, promoting that of arginase/nitric oxide synthase (ARG/NOS) pathway^20^, and suppressing that of poly(ADP-Ribose) polymerase 1 (PARP1) signaling pathway^21^. Following PSKα application, there is sufficient NADPH and ATP (intracellular reducing power and energy currency) provision^18,21^, which increases the activities of reactive oxygen species (ROS) avoiding and scavenging systems^17,18^. This activates the accumulation of endogenous melatonin^15^, hydrogen sulfide^22^, cytokinin^19^, γ-aminobutyric acid (GABA), proline, nitric oxide, and polyamines^20^, enhancing the activities of intracellular molecular chaperone and protein repairing systems^17,19^, suppressing ethylene biosynthesis and chlorophyll degradation^22^, improving ABTS, FRAP, and DPPH scavenging capacity due to the accumulation of phenols, flavonoids, anthocyanins, and ascorbic acid^15,16^. These help preserve membrane integrity as realized by lower electrolyte leakage and malondialdehyde (MDA) accumulation, which might be accountable for palliating chilling injury and fungal decay, postponing senescence, and preserving sensory and nutritional quality in fruits and vegetables during postharvest life^23^.

This study elucidates the endogenous SUMO1/SUMO E3 ligase SIZ1 signaling pathway during bud opening and petal senescence of rose flowers. Furthermore, the potential of PSKα treatment for delaying senescence and prolonging vase life of cut rose flowers was evaluated by studying (1) *SUMO1* and *SUMO E3 ligase SIZ1* genes expression, (2) endogenous accumulation of H_2_O_2_ by gene expression and activities of superoxide dismutase (*SOD*), catalase (*CAT*), ascorbate peroxidase (*APX*), and glutathione reductase (*GR*); (3) endogenous accumulation of proline by gene expression and activities of pyrroline-5-carboxylate synthase (*P5CS*) and ornithine aminotransferase (*OAT*); (4) activity of endogenous GABA shunt pathway by gene expression and activities of glutamate decarboxylase (*GAD*), GABA transaminase (*GABA-T*), and succinate semialdehyde dehydrogenase (*SSADH*) as well as the expression of heat shock proteins 70/90 (*HSP70/90*).

## Materials and methods

### SUMO E3 ligase SIZ1 signaling pathway during bud opening and petal senescence of rose flowers

For illustrating the SUMO E3 ligase SIZ1 signaling pathway during bud opening and petal senescence, commercial cut rose flowers (*Rosa hybrida* cv. Angelina) were obtained from a legalized local commercial greenhouse in Qazvin Province, Iran. All protocols were complied with relevant institutional, national, and international guidelines and legislation. For developmental stage 1, closed buds; stage 2, closed and heavily pigmented bud; and stage 3, flowers with their outer petal whorl just unfurled as commercial harvest stage, outermost-to-innermost petals from 10 flowers in three replications (30 flowers, 10 flowers per replication) were harvested according to Aghdam et al.^1^. After harvesting at the commercial stage (stage 3), flowers were re-cut underwater to a stem length of 40 cm to avoid air embolism, with six leaves. Flowers were placed randomly in 1000 mL distilled water for 12 days in three replications (30 flowers, 10 flowers per replication). During vase life at 20 ± 1 °C and 60 ± 5% relative humidity, petals were harvested from the flowers at stage 4, sepals completely opened, petal starting to unfold (flowers 4 days after harvest); stage 5, petals completely unfolded (flowers 8 days after harvest); and stage 6, flowers completely senesced with petal bluing (flowers 12 days after harvest). The vase solution was replaced with fresh distilled water every day. For developmental stages 4–6, outermost-to-innermost petals from 10 flowers were harvested, according to Aghdam et al.^1^.

### Rose flowers and PSKα treatment

Regarding longest vase life, 16 days for PSKα at 150 nM in comparison with 12 days for PSKα at 0 nM as reported by Aghdam et al.^1^, 180 flowers (*Rosa hybrida* cv. Angelina) harvested at commercial-stage (stage 3), was re-cut underwater to a stem-length of 40 cm to avoid air embolism, with six leaves. Flowers were placed randomly in 1000 mL distilled water or PSKα at 150 nM for 24 h as pulse treatment. PSKα (soluble in sterile water; 1 mg/mL) was provided by Pepmic Co., Ltd, Suzhou (China). After pulse treatment for 24 h, the vase solution was replaced with fresh distilled water for 4, 8, and 12 days, in three replications, respectively (90 flowers, 30 flowers per replication, 10 flowers per sampling time). During vase life at 4, 8, and 12 days after harvest, outermost-to-innermost petals from 10 flowers were harvested, according to Aghdam et al.^1^. During developmental stages and vase life, petals harvested from flowers were immediately frozen in liquid nitrogen, powdered, and stored at − 80 °C for biochemical and gene expression analysis.

### Activities of SOD, CAT, APX, and GR and endogenous H_2_O_2_ accumulation

To analyze the SOD, CAT, APX, and GR activities, five grams of the frozen powder was homogenized with 25 mL of phosphate buffer (50 mM; pH 7.8) containing 0.2 mM EDTA and 2% PVP. SOD, CAT, APX, and GR enzymes activity were determined according to Giannopolitis and Ries^24^, Beers and Sizer^25^, Nakano and Asada^26^, and Sofo et al.^27^, respectively. For CAT activity, 100 μL of the enzyme extract was added to 2.9 mL of reaction mixture containing 15 mM H_2_O_2_ and 50 mM phosphate buffer (pH 7). The degradation of H_2_O_2_ was measured by the decrease of absorbance at 240 nm during 1 min. One unit of CAT activity was defined as a decrease in absorbance at 240 nm of 0.01 per min. For APX activity, 100 μL of the enzyme extract was added to 2.9 mL of reaction mixture containing 50 mM phosphate buffer (pH 7), 0.5 mM ascorbic acid and 1 mM H_2_O_2_. The decrease of absorbance at 290 nm during 1 min was measured. One unit of APX activity was defined as the enzyme that oxidizes 1 μmol of ascorbate per minute. For SOD activity, 100 μL of enzyme extract was added to 2.9 mL of reaction mixture containing 50 mM phosphate buffer (pH 7), 5 mM methionine, 100 μM EDTA, 65 μM NBT, and 40 μL of 0.15 mM riboflavin. The tubes were then placed in a fluorescent light incubator (40 W, 10 min), and the formation of blue formazan was monitored by recording the absorbance at 560 nm. One unit of SOD activity is defined as the enzyme that causes a 50% inhibition of NBT reduction under assay conditions. For GR activity based on the decrease in absorbance at 340 nm due to NADPH oxidation, 200 μL of enzyme extract was added to a reaction mixture containing 1.5 mL of 0.1 M phosphate buffer (pH 7), 150 μL of 20 mM GSSG, 1 mL of distilled water and 150 μL of 2 mM NADPH (dissolved in Tris–HCl buffer, pH 7), in a final volume of 3.0 mL. One unit of GR activity was defined as the enzyme that oxidizes 1 nmol of NADPH per min at 25 °C. Their activities were expressed as U kg^−1^ protein. The endogenous H_2_O_2_ accumulation was assayed following titanium (IV) procedure according to Patterson et al.^28^. One gram of the frozen powder was homogenized with 5 mL of acetone at 0 °C. After centrifugation for 15 min at 6000×g at 4 °C, the supernatant was collected. The supernatant (1 mL) was mixed with 0.1 mL of 5% titanium sulfate and 0.2 mL ammonia and then centrifuged for 10 min at 6000×g at 4 °C. The pellets were dissolved in 3 mL of 10% (v/v) H_2_SO_4_ and centrifuged for 10 min at 5000×g at 4 °C. The absorbance of the supernatant was measured at 410 nm. The endogenous H_2_O_2_ accumulation was expressed as μmol kg^−1^ on a fresh weight basis.

### Activities of P5CS and OAT and endogenous proline accumulation

According to Zhang et al. (2013), the endogenous proline accumulation was measured following the acid ninhydrin method. One gram of the frozen powder was homogenized in 5 mL of 3% (v/v) sulfosalicylic acid and centrifuged at 12,000×g for 10 min. Two mL of glacial acetic acid and 3 mL of ninhydrin reagent were mixed with supernatant (2 mL) and boiled for 10 min. Then 4 mL of toluene was added into the reaction mixture after the solution was cooled. The absorbance of the organic phase was recorded at 520 nm. The endogenous proline accumulation was expressed as mmol kg^−1^ on a fresh weight basis. For OAT activity, according to Shan et al.^29^, the reaction mixture contained 35 mM l-ornithine, 0.05 mM PLP, 25 mM a-ketoglutarate, and the enzyme extract. The mixture was incubated at 37 °C for 20 min before adding 3M HClO_4_. Then, 2% ninhydrin was added into the mixture and boiled for 20 min. After cooling, the mixture was centrifuged at 15,000×g for 10 min. The residue was dissolved in 2 mL toluene and quantified by measuring the absorbance at 510 nm. For P5CS activity according to Shan et al.^29^, the reaction mixture contained 100 mM Tris–HCl (pH 7.2), 25 mM MgCl_2_, 75 mM sodium glutamate, 5 mM ATP, 0.4 mM NADPH and 200 μL enzyme extract. One unit of P5CS and OAT enzymes activity was defined as an enzyme that caused a decrease by 0.001 in an absorbance per minute at 340 nm. P5CS and OAT activity were expressed as U kg^−1^ protein.

### Activities of GAD, GABA-T, and SSADH and endogenous GABA accumulation

The enzymatic assay with GABase was employed to quantify GABA accumulation described by Deewatthanawong et al.^30^. For endogenous GABA accumulation assessment, one gram of the frozen powder was homogenized in 5 mL of methanol for 10 min at room temperature. After vacuum dried, dissolved in 1 mL 70 mM lanthanum chloride followed by 15 min of shaking, and centrifugation at 13,000×g for 15 min. Then, 800 μL supernatant was mixed with 160 μL 1M KOH. After being shaken for 5 min, and centrifugation at 13,000×g for 10 min, the supernatant was used for GABA determination. The GABase assay mixture contained 75 mM potassium pyrophosphate (pH 8.6), 3.3 mM β-mercaptoethanol, 10 mM 2-oxoglutarate, 1.25 mM NADP^+^, and 0.016-unit GABase. The absorbance at 340 nm was read before, and 10 min after adding α-ketoglutarate using the 96-well plate reader, and GABA was determined by comparison with a standard curve of GABA. GABA accumulation was expressed as mmol kg^−1^ fresh weight.

GAD, GABA-T, and SSADH activity were determined according to Bartyzel et al.^31^, Ansari et al.^32^, and Thorburn et al.^33^. For GAD, GABA-T and SSADH extraction, two grams of the frozen powder was homogenized in 12 mL of 0.1 M Tris–HCl buffer (pH 9.1) containing 5 mM EDTA, 1 mM phenylmethylsulfonyl fluoride (PMSF), 1 mM dithiothreitol (DTT), 10% (v/v) glycerol, 0.5 mM pyridoxal phosphate (PLP) and 0.1% polyvinylpolypyrrolidone (w/v) (PVPP). The homogenate was centrifuged at 10,000×g for 15 min at 4 °C and the supernatant was filtered and concentrated with Amicon Ultra centrifugal filters 10,000 MWCO (Millipore®). Then, it was re-suspended in 1.5 mL of 0.2 M sodium phosphate buffer (pH 5.8) containing 0.04 mM PLP for GAD activity, or in 1.5 mL of 50 mM Tris–HCl buffer (pH 8.2) containing 0.75 mM EDTA, 1.5 mM DTT, 10% (v/v) glycerol, 0.2 mM PLP for GABA-T activity or in 1.5 mL of 0.1M sodium phosphate buffer (pH 9) containing 1 mM DTT and 1 mM EDTA for SSADH activity. Activities of GAD, GABA-T, and SSADH enzymes were analyzed based on the production of GABA, alanine, and succinate and expressed as μmol GABA or alanine or succinate kg^−1^ protein min^−1^.

### Gene expression analysis by quantitative real-time PCR

Total RNA was isolated from petals using the RNeasy Plant Mini Kit (Qiagen, Hilden, Germany). Then, gel electrophoresis by 1% agarose gel and absorbance at 260 nm by NanoDrop spectrophotometer (BioTek, EPOCH, serial 121004C, USA) was used for evaluating RNA quality and quantity. After RNase-free DNaseI (Thermo Fisher Scientific) treatment, one µg RNA was applied to synthesize the first-strand cDNA HyperscriptTM Reverse Transcriptase (GeneAll Inc, South Korea). Real-time quantitative PCR was fulfilled by BioRad system (Bio-Rad, Hercules, CA, USA) using the SYBR^®^Green PCR Master Mix 2X (Amplicon, Denmark). The primers used to amplify the gene sequences are listed in Table 1. The threshold cycle (Ct) value was normalized to Actin Ct value as a housekeeping gene^1^. The relative expression of the gene was calculated by formula 2^−ΔΔCt^ according to Livak and Schmittgen^34^.

**Table 1:** Primers used for qRT-PCR.

Gene name	Accession numbers	Functional annotations	Primer sequences (5′–3′)
*SUMO1*	XM024336892	Protein SUMOylation	F: TCTTCGGGCCTTTAGAACCT R: TCAGTTCAGGGTTTGGGTTT
*SIZ1*	XM040519042	Protein SUMOylation	F: GCTGAACCTGCAGACACAAA R: CAGATACACTCGCGGTCTCA
*SOD*	KJ159890	ROS scavenging	F: ATCGTGGATAGCCAGATACCA R: CAACCACACCACATGCCAATC
*CAT*	MF667950	ROS scavenging	F: TGTGCACACCTACCGAATGA R: CATCAGCTCCTCGCATCGTC
*APX*	KY274158	ROS scavenging	F: TTGTGCTCTACTCGTGCCAG R: TGACGGTTGGGTAGCACTTC
*GR*	MF667955	ROS scavenging	F: GTCATCCGCATGGAGCACTA R: GCACAGGTCTCGAAAGTCCA
*P5CS*	XM024334137	Proline biosynthesis	F: AGCAATAAACTGGTTTCTCAA R: GCCATATCCATATTAGCAGAC
*OAT*	XM024318202	Proline biosynthesis	F: GATGAAATACAATCTGGTTTAGC R: CCGCACTTACAGGTATCA
*GAD*	XM024333661	GABA shunt pathway	F: GAGGTGAAGTTGAGGGATGATT R: CATTAAGAGTGGAACCGAGGATAG
*GABA-T*	XR005804965	GABA shunt pathway	F: AGAAGTCAGAGGGTAGCTATGT R: CCACTAGTCGAGGCTCATTTC
*SSADH*	XM024304033	GABA shunt pathway	F: GCAAGTCCGCAGGTAAGAAA R: TGCCACCAAGTTCAAGAGATAC
*HSP70*	XM024321184	Molecular chaperones	F: GTGCTACAAGGTGAGCGTGA R: GCTCTGTCCTTGGCAGAAAC
*HSP90*	XP024185377	Molecular chaperones	F: GGAAGTGTAACCCTGCCAAA R: CAAGCTCTCGAAGGAACACC
*Actin*	AF044573	Housekeeping gene	F: CCATGAGACCACATACAATTCG R: CAGCAGTAAGCCTACAAGGTCATA

### Statistical analysis

The experiment was planned using a factorial design with three biological replications and two factors: PSKα treatments (0 and 150 nM PSKα) and vase life duration (4, 8, and 12 days). Three technical replications were carried out by three extractions from each biological replication for biochemical and gene expression analyses to avoid the instrumental error. The means of three technical replications are considered as one biological replication. All analyses were performed by using SPSS software^35^ version 21 (IBM SPSS: Chicago, IL, USA). The heat map of genes expression in rose flowers during opening and senescence was created in R using the Pheatmap package^36^. All data were expressed as mean ± standard error (SE) from three biological replications. Fisher’s least significant differences (LSD) tests were performed to compare differences between mean values at *P* < 0.05.

## Results

### SUMO1/SUMO E3 ligase SIZ1 signaling pathway during flower bud opening

**Figure 1:**
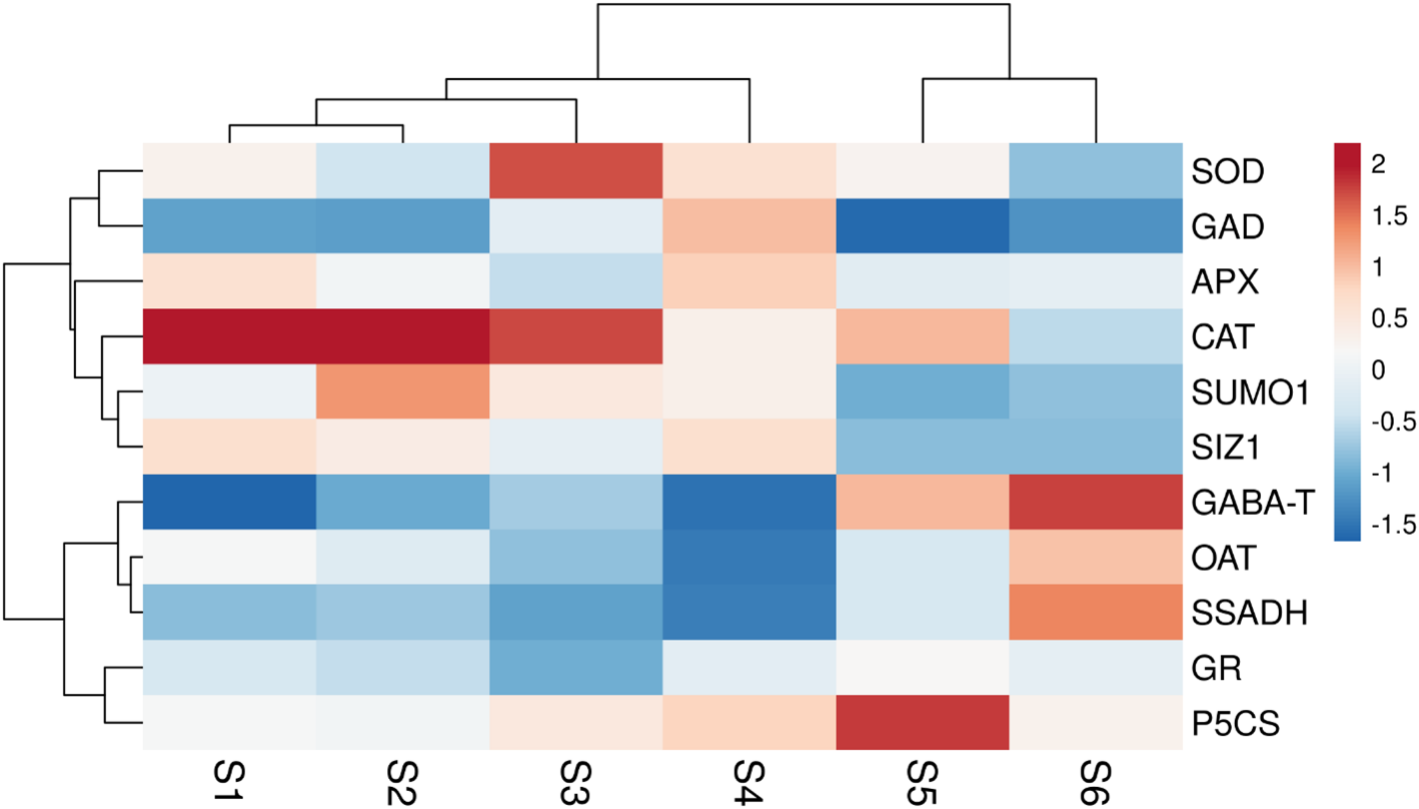
Heat map was created in R using the pheatmap package for representing *SUMO1/SIZ1* accompanying ROS scavenging, proline biosynthesis and GABA shunt pathway genes expression in rose flowers during opening and senescence. Stage 1, closed buds; stage 2, closed and heavily pigmented bud; and stage 3, flower with their outer petal whorl just unfurled as commercial harvest stage, stage 4, sepals completely opened, petal starting to unfold (4 days after harvest); stage 5, petals completely unfolded (8 days after harvest); and stage 6, flower completely senesced with petal bluing (12 days after harvest).

As shown in Fig. 1, rose flowers exhibited higher expression of *SUMO1* and *SUMO E3 ligase SIZ1* (*P* < 0.01) during bud opening. Matching with higher SUMO1/SUMO E3 ligase SIZ1 genes expression, higher expression and activities (*P* < 0.01) of *SOD*, *CAT*, *APX*, and *GR* were associated with lower endogenous H_2_O_2_ accumulation (*P* < 0.01) in rose flowers during bud opening (Fig. 1; Table 2). In addition, higher endogenous proline (Table 3) and GABA accumulation (Table 4) (*P* < 0.01) in rose flowers during bud opening was concomitant with higher expression and activity *P5CS* and *GAD*, respectively (Fig. 1; Tables 3 and 4) (*P* < 0.01).

**Table 2:** SOD, CAT, APX and GR activity accompanying by endogenous H_2_O_2_ accumulation during rose flowers development from bud opening to petal senescence.

Flower development stages	SOD activity (U kg^−1^)	CAT activity (U kg^−1^)	APX activity (U kg^−1^)	GR activity (U kg^−1^)	H_2_O_2_ accumulation (μmol kg^−1^ FW)
Stage 1	35.29 ± 2.37 d	88.18 ± 5.14 b	18.97 ± 1.30 c	12.36 ± 1.01 c	15.60 ± 0.84 c
Stage 2	43.36 ± 3.35 d	115.50 ± 7.79 a	22.47 ± 1.62 c	17.87 ± 1.85 c	19.71 ± 2.09 c
Stage 3	69.42 ± 5.37 c	128.74 ± 6.03 a	48.73 ± 4.23 b	38.34 ± 3.45 a	22.80 ± 1.93 c
Stage 4	128.74 ± 5.73 a	113.33 ± 7.37 a	69.83 ± 5.64 a	44.99 ± 2.90 a	36.04 ± 2.41 b
Stage 5	94.48 ± 3.59 b	78.51 ± 3.80 b	42.87 ± 4.38 b	29.20 ± 3.30 b	86.29 ± 4.59 a
Stage 6	46.82 ± 3.76 d	50.18 ± 2.77 c	28.70 ± 3.14 c	17.53 ± 0.94 c	89.01 ± 4.46 a
Significant	**	**	**	**	**
CV	10.42	10.44	16.69	16.01	11.74
LSD (*P* < 0.05)	12.92	17.78	11.46	7.61	9.38

Data shown are mean values of n = 3, and the error bars represent standard errors of the means. Mean values followed by different letters indicate they are different by LSD test at *P* < 0.05.

**Table 3:** P5CS and OAT activity accompanying by proline accumulation during rose flowers development from bud opening to petal senescence.

Flower development stages	P5CS activity (U kg^−1^)	OAT activity (U kg^−1^)	Proline accumulation (mmol kg^−1^ FW)
Stage 1	11.93 ± 0.81 c	8.25 ± 0.61 d	15.46 ± 0.34 e
Stage 2	15.37 ± 1.42 bc	11.28 ± 0.93 d	26.57 ± 1.55 d
Stage 3	22.33 ± 1.84 b	13.27 ± 0.49 cd	38.97 ± 3.40 c
Stage 4	38.97 ± 3.82 a	17.26 ± 1.15 c	55.86 ± 3.59 b
Stage 5	38.62 ± 3.83 a	39.32 ± 3.19 a	69.13 ± 4.79 a
Stage 6	33.07 ± 2.21 a	32.64 ± 1.96 b	54.51 ± 2.70 b
Significant	**	**	**
CV	16.79	14.24	12.31
LSD (*P* < 0.05)	7.98	5.15	9.51

Data shown are mean values of n = 3, and the error bars represent standard errors of the means. Mean values followed by different letters indicate they are different by LSD test at *P* < 0.05.

**Table 4:** GAD, GABA-T and SSADH activity accompanying by GABA accumulation during rose flowers development from bud opening to petal senescence.

Flower development stages	GAD activity (μmol kg^−1^ min^−1^)	GABA-T activity (μmol kg^−1^ min^−1^)	SSADH activity (μmol kg^−1^ min^−1^)	GABA accumulation (mmol kg^−1^ FW)
Stage 1	25.86 ± 2.34 d	19.26 ± 1.40 d	9.26 ± 0.74 e	18.53 ± 0.56 d
Stage 2	38.52 ± 3.29 bc	22.46 ± 1.58 cd	13.51 ± 0.87 de	22.46 ± 1.55 cd
Stage 3	55.16 ± 2.95 a	27.41 ± 2.08 bc	17.43 ± 1.29 cd	29.08 ± 3.73 bc
Stage 4	60.14 ± 4.79 a	24.37 ± 1.29 cd	22.71 ± 1.54 c	39.56 ± 3.96 a
Stage 5	42.84 ± 1.82 b	31.24 ± 2.53 b	33.21 ± 1.94 b	33.05 ± 1.90 ab
Stage 6	29.52 ± 2.31 cd	49.45 ± 3.54 a	42.69 ± 3.92 a	28.88 ± 2.40 bc
Significant	**	**	**	**
CV	12.66	13.20	15.13	15.95
LSD (*P* < 0.05)	9.46	6.81	6.23	8.11

Data shown are mean values of n = 3, and the error bars represent standard errors of the means. Mean values followed by different letters indicate they are different by LSD test at *P* < 0.05.

### SUMO1/SUMO E3 ligase SIZ1 signaling pathway during petal senescence

Harvested rose flowers exhibited lower expression of *SUMO1* and *SUMO E3 ligase SIZ1* (Fig. 1; *P* < 0.01). Matching with lower SUMO1/SUMO E3 ligase SIZ1 signaling pathway, lower expression and activities of *SOD*, *CAT*, *APX*, and *GR* (*P* < 0.01) were associated with higher endogenous H_2_O_2_ accumulation (*P* < 0.01) in cut rose flowers during petal senescence (Fig. 1; Table 2). Also, cut rose flowers during petal senescence exhibited lower expression and activity of *P5CS* (*P* < 0.01) concomitant with higher expression and activity of *OAT* (*P* < 0.01), thereby activating endogenous proline accumulation (*P* < 0.01) in cut rose flowers during petal senescence (Fig. 1; Table 3). In addition, lower GABA accumulation in cut rose flowers during petal senescence was associated with higher expression and activities of *GABA-T* and *SSADH* (*P* < 0.01) (Fig. 1; Table 4).

### PSKα treatment and petal senescence in cut rose flowers

**Figure 2:**
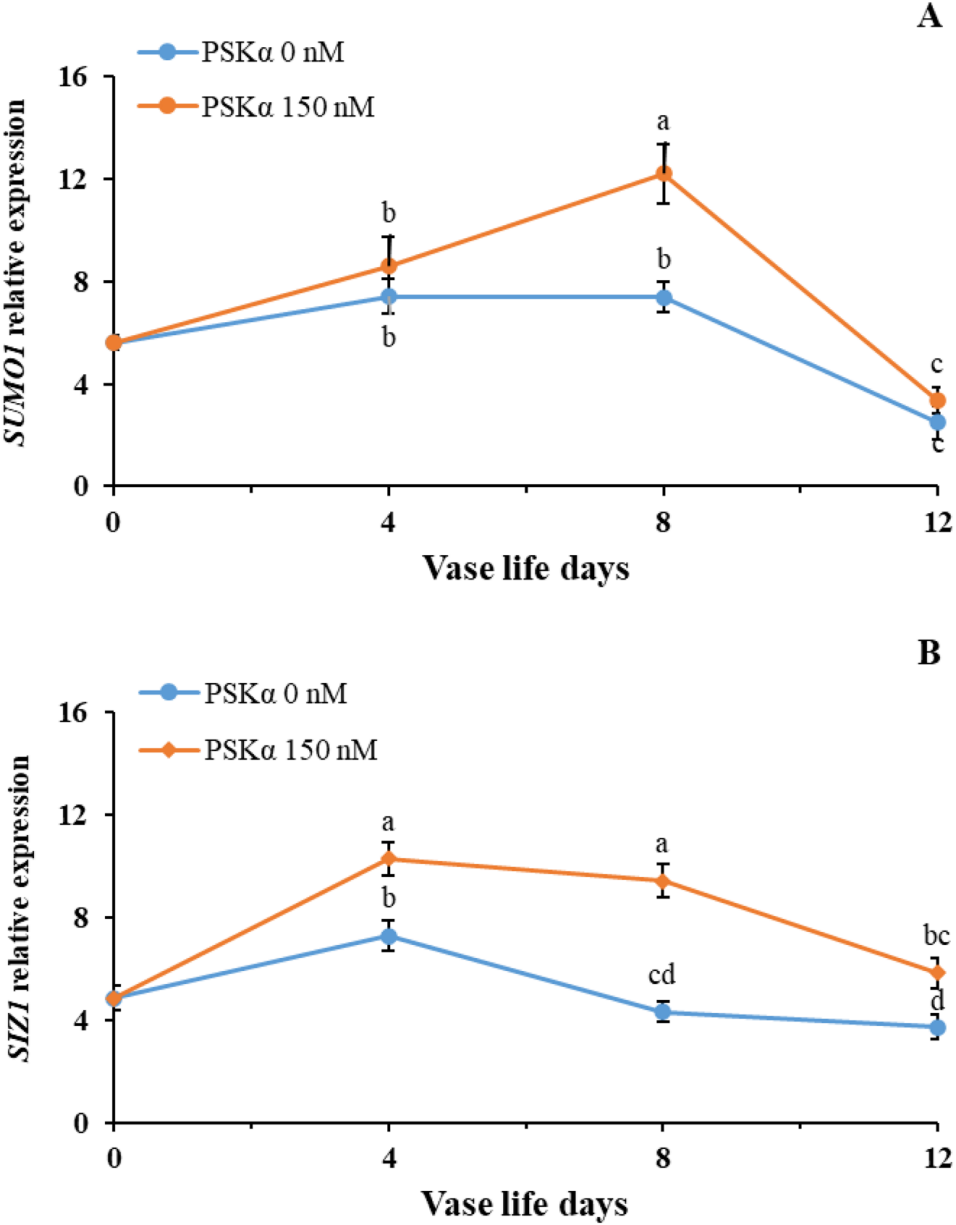
*SUMO1* (**A**) and *SUMO E3 ligase SIZ1* (**B**) genes expression in petals of rose flowers treated with PSKα at 0 and 150 nM during vase life at 20 °C for 12 days. Data shown are mean values of n = 3, and the error bars represent standard errors of the means. Mean values followed by different letters indicate they are different by LSD test at *P* < 0.05.

**Figure 3:**
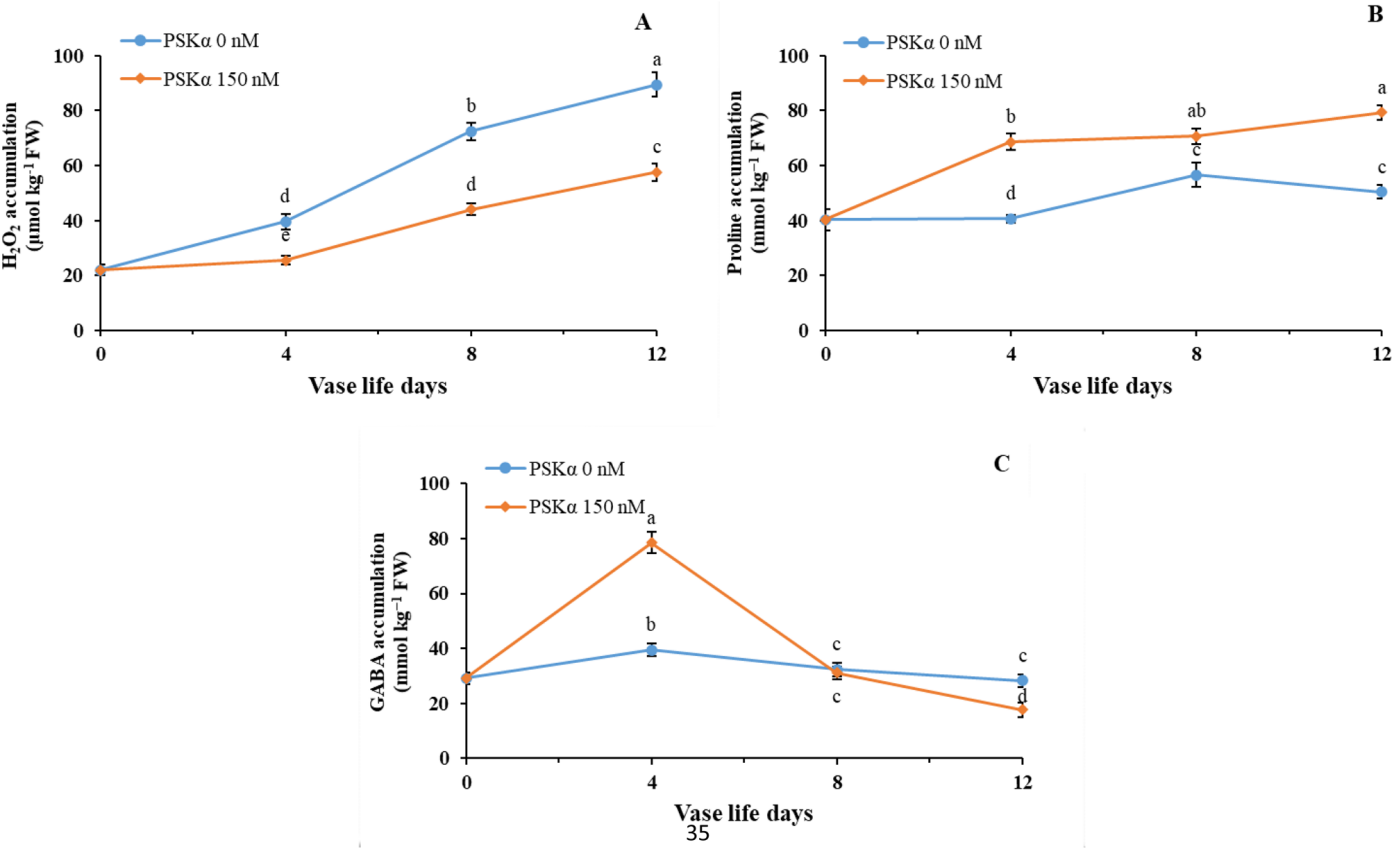
Endogenous H_2_O_2_ (**A**), proline (**B**) and GABA (**C**) accumulation in petals of rose flowers treated with PSKα at 0 and 150 nM during vase life at 20 °C for 12 days. Data shown are mean values of n = 3, and the error bars represent standard errors of the means. Mean values followed by different letters indicate they are different by LSD test at *P* < 0.05.

**Figure 4:**
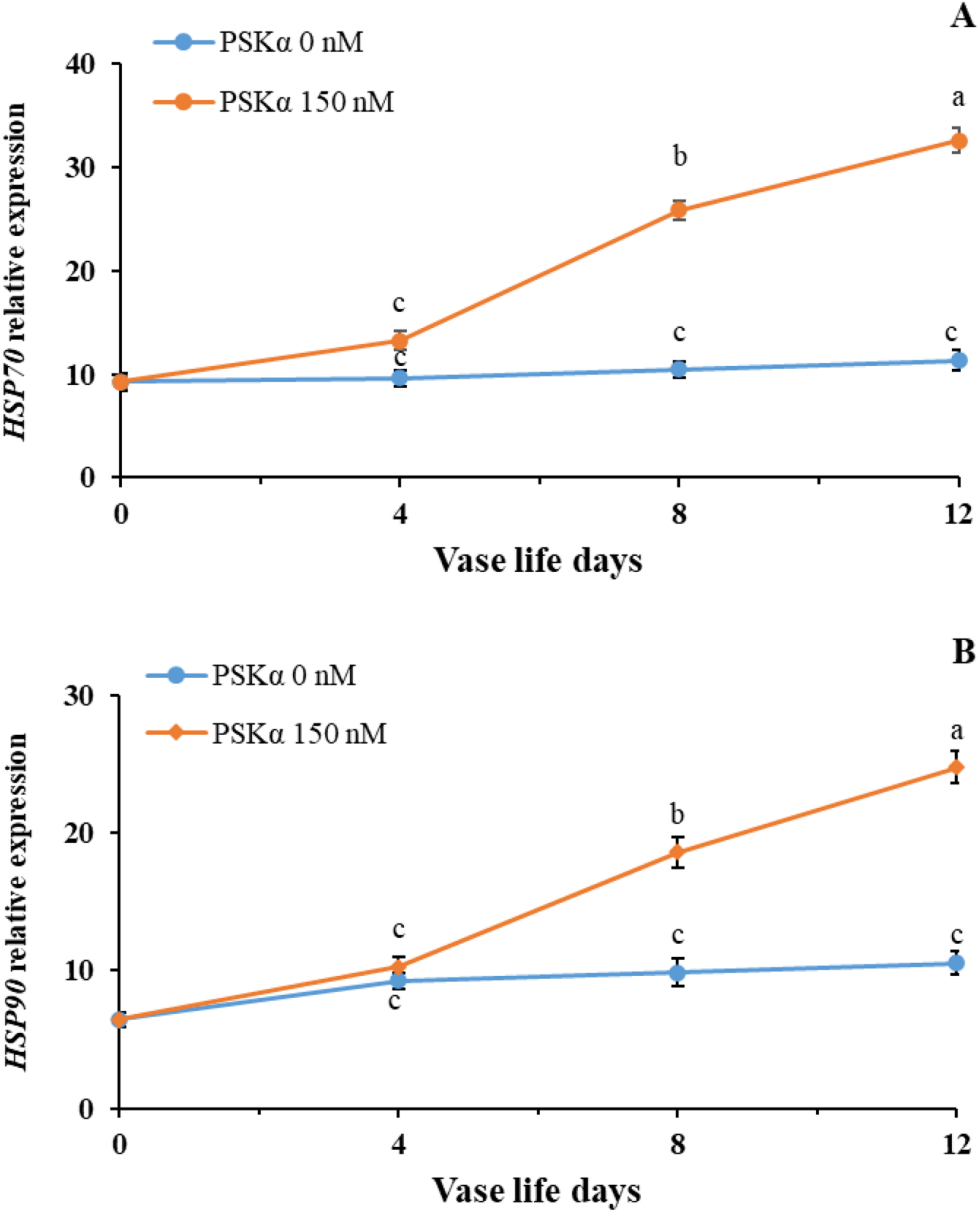
*HSP70* (**A**) and *HSP90* (**B**) genes expression in petals of rose flowers treated with PSKα at 0 and 150 nM during vase life at 20 °C for 12 days. Data shown are mean values of n = 3, and the error bars represent standard errors of the means. Mean values followed by different letters indicate they are different by LSD test at *P* < 0.05.

As shown in Fig. 2, reinforcing the expression of *SUMO1* and *SUMO E3 ligase SIZ1* (*P* < 0.01) by 150 nM PSKα treatment might postpone senescence, thus extending vase life in cut rose flowers. Matching with higher *SUMO1* and *SUMO E3 ligase SIZ1* genes expression, the lower endogenous H_2_O_2_ accumulation (Fig. 3A; *P* < 0.01) in cut rose flowers after the administration of 150 nM PSKα treatment might be attributed to higher expression and activities of *SOD*, *CAT*, *APX*, and *GR* (Table 5; *P* < 0.01). As shown in Fig. 3B, higher endogenous proline accumulation (*P* < 0.01) in cut rose flowers following the administration of 150 nM PSKα treatment might be attributed to higher expression and activities of *P5CS* and *OAT* (Table 6; *P* < 0.01). Furthermore, 150 nM PSKα treatment promoted GABA utilizing (Fig. 3C) by the activity of GABA shunt pathway as realized by higher expression and activities of *GAD*, *GABA-T*, and *SSADH* (Table 7; *P* < 0.01) in cut rose flowers. Moreover, higher expression of *HSP70* and *HSP90* (Fig. 4A,B; *P* < 0.01) in cut rose flowers following the administration of 150 nM PSKα treatment might be accountable for postponing senescence (Supplementary Fig. 1).

**Table 5:** *SOD*, *CAT*, *APX* and *GR* genes expression and enzymes activity in petals of rose flowers treated with PSKα at 0 and 150 nM during vase life at 20 °C for 12 days.

PSKα treatment (nM)	Vase life (days)	Genes relative expression				Enzyme’s activity (U kg^−1^)			
		*SOD*	*CAT*	*APX*	*GR*	SOD	CAT	APX	GR
0	0	6.37 ± 0.85	7.23 ± 0.57	3.78 ± 0.34	3.25 ± 0.29	58.60 ± 4.36	128.73 ± 5.40	49.42 ± 4.85	37.52 ± 6.26
	4	8.66 ± 0.71 bc	5.95 ± 0.52 cd	8.67 ± 0.38 bc	6.51 ± 0.41 b	85.58 ± 2.5 b	110.53 ± 2.67 b	72.35 ± 2.33 c	42.38 ± 2.42 b
	8	8.31 ± 0.64 bc	7.41 ± 0.80 c	7.34 ± 0.52 cd	8.16 ± 0.66 b	72.26 ± 1.14 c	72.26 ± 2.42 d	39.55 ± 1.84 e	33.25 ± 1.67 c
	12	3.47 ± 0.27 d	2.87 ± 0.39 e	5.54 ± 0.61 d	3.65 ± 0.28 c	36.23 ± 1.09 e	29.90 ± 1.77 e	28.35 ± 1.29 f.	16.22 ± 0.91 d
150	4	10.39 ± 0.98 ab	10.32 ± 0.66 b	12.33 ± 1.11 a	7.28 ± 0.49 b	98.56 ± 4.10 a	136.55 ± 5.07 a	95.45 ± 2.67 a	48.15 ± 3.40 ab
	8	12.30 ± 1.63 a	13.74 ± 1.41 a	10.60 ± 0.79 ab	11.39 ± 1.28 a	103.09 ± 4.14 a	98.87 ± 4.79 c	82.55 ± 3.54 b	52.61 ± 3.95 a
	12	6.52 ± 0.47 c	4.85 ± 0.27 de	6.33 ± 0.34 d	8.45 ± 0.62 b	59.70 ± 1.89 d	39.49 ± 2.45 e	47.49 ± 1.65 d	40.43 ± 2.65 bc

Significant	df								
Treatment	1	**	**	**	**	**	**	**	**
Time	2	**	**	**	**	**	**	**	**
T × T	2	ns	*	ns	*	*	*	**	*
CV	–	18.81	17.70	13.86	16.01	6.35	7.31	6.64	12.02
LSD (*P* = 0.05)		2.77	2.37	2.09	2.16	8.57	10.58	7.21	8.31

Data shown are mean values of n = 3, and the error bars represent standard errors of the means. Mean values followed by different letters indicate they are different by LSD test at *P* < 0.05.

**Table 6:** *P5CS* and *OAT* genes expression and enzymes activity in petals of rose flowers treated with PSKα at 0 and 150 nM during vase life at 20 °C for 12 days.

PSKα treatment (nM)	Vase life (days)	Genes relative expression		Enzyme’s activity (μmol kg^−1^ min^−1^)	
		*P5CS*	*OAT*	P5CS	OAT
0	0	5.83 ± 0.57	3.80 ± 0.49	22.82 ± 1.23	13.57 ± 1.40
	4	8.42 ± 1.02 b	3.97 ± 0.37 c	25.33 ± 1.76 c	15.21 ± 0.62 d
	8	9.58 ± 0.83 b	4.61 ± 0.36 c	37.85 ± 2.97 b	38.41 ± 0.98 b
	12	3.63 ± 1.38 c	7.45 ± 0.70 b	12.36 ± 1.04 d	15.73 ± 0.65 d
150	4	14.48 ± 1.56 a	7.57 ± 0.52 b	42.69 ± 2.45 b	22.13 ± 1.08 c
	8	15.52 ± 1.79 a	10.47 ± 0.58 a	74.47 ± 3.18 a	55.05 ± 2.92 a
	12	9.50 ± 0.73 b	9.66 ± 0.60 a	39.30 ± 2.46 b	39.54 ± 1.60 b

Significant	df				
Treatment	1	**	**	**	**
Time	2	**	**	**	**
T × T	2	ns	*	**	**
CV	–	21.76	12.79	10.85	8.54
LSD (*P* = 0.05)		3.94	1.66	7.46	4.71

Data shown are mean values of n = 3, and the error bars represent standard errors of the means. Mean values followed by different letters indicate they are different by LSD test at *P* < 0.05.

**Table 7:** *GAD*, *GABA-T* and *SSADH* genes expression and enzymes activity in petals of rose flowers treated with PSKα at 0 and 150 nM during vase life at 20 °C for 12 days.

PSKα treatment (nM)	Vase life (days)	Genes relative expression			Enzyme’s activity (μmol kg^−1^ min^−1^)		
		*GAD*	*GABA-T*	*SSADH*	GAD	GABA-T	SSADH
0	0	4.26 ± 0.28	3.57 ± 34	2.96 ± 0.31	54.67 ± 4.42	27.48 ± 1.46	17.20 ± 1.21
	4	10.04 ± 0.61 b	3.98 ± 0.25 d	4.34 ± 0.14 c	59.26 ± 3.60 b	30.78 ± 1.54 c	21.46 ± 0.65 d
	8	5.33 ± 0.62 cd	5.32 ± 0.35 c	7.50 ± 0.27 b	42.35 ± 1.32 c	32.29 ± 1.65 c	32.37 ± 1.61 c
	12	2.64 ± 0.18 d	6.61 ± 0.35 b	8.55 ± 0.26 b	29.38 ± 0.97 d	51.73 ± 0.94 ab	42.69 ± 1.66 b
150	4	16.19 ± 1.74 a	4.28 ± 0.16 d	5.28 ± 0.21 c	67.69 ± 1.92 a	35.37 ± 1.61 c	26.39 ± 0.78 d
	8	7.74 ± 0.75 bc	7.41 ± 0.34 b	10.77 ± 0.88 a	62.66 ± 1.51 ab	46.98 ± 1.74 b	46.69 ± 2.24 b
	12	3.52 ± 0.83 d	10.39 ± 0.44 a	11.83 ± 0.64 a	48.89 ± 3.40 c	57.62 ± 3.25 a	54.07 ± 2.30 a

Significant	df						
Treatment	1	**	**	**	**	**	**
Time	2	**	**	**	**	**	**
T × T	2	*	**	*	*	*	*
CV	–	21.01	9.00	10.36	7.88	7.84	7.75
LSD (*P* = 0.05)			1.01	1.48	7.25	5.92	5.14

Data shown are mean values of n = 3, and the error bars represent standard errors of the means. Mean values followed by different letters indicate they are different by LSD test at *P* < 0.05.

## Discussion

During rose flower bud opening, glucose supply from light and CO_2_ dependent photosynthesis might be accountable for increasing TOR signaling pathway by providing energy relay through glycolysis pathway, tricarboxylic acid cycle, and electron transporting system^37^. In addition, providing sulfide (S_2_^−^) from sulfate (SO_4_^2−^) assimilation mediated by sulfite reductase (SiR) enzyme might be accountable for increasing TOR signaling pathway by increasing glucose supply and promoting the activity of the glucose/energy signaling pathway^38^. In addition, glucose and light-dependent auxin biosynthesis may trigger the TOR signaling pathway by activating ROP2 GTPase^39^. In addition to glucose and light-dependent auxin biosynthesis, ROP2 GTPase might be activated by sufficient nitrate (NO_3_^−^) and ammonium (NH_4_^+^) to trigger TOR signaling pathway. In addition, providing glutamine (Gln) from nitrogen assimilation, delivering cysteine (Cys) from sulfur assimilation, and providing glycine (Gly) from the glycolate pathway exhibit the highest potency for increasing TOR signaling pathway by activating ROP2 GTPase^40–42^. Moreover, increasing glucose supply and promoting the activity of the glucose/energy signaling pathway is not accountable for increasing TOR signaling pathway following nitrate-ammonium supplying^40–42^. In plants, the TOR signaling pathway might be accountable for increasing plant growth-promoting auxin (IAA), gibberellic acid (GA3), brassinosteroids (BRs), and cytokinins (CKs) signaling while suppressing plant growth-inhibiting abscisic acid (ABA), jasmonic acid (JA), salicylic acid (SA), and ethylene (ETH) signaling^42^. By activating endogenous GA3 biosynthesis and signaling from the TOR signaling pathway, petal cell expansion, hypertrophy, or enlargement is promoted by the accumulation of vacuolar sugars, cell wall loosening or relaxation, and vacuolar water flows^43,44^. In addition, promoting the degradation of starch and sucrose through the TOR signaling pathway might be accountable for the greater decorative quality of rose flowers during bud opening because it provides sufficient intracellular glucose 6-phosphate used to supply sufficient intracellular (1) ATP through glycolysis pathway, tricarboxylic acid cycle, and electron transporting system, and (2) NADPH, erythritol-4-phosphate, and ribose 5-phosphate through oxidative pentose phosphate pathway^37,43,44^. Following the exogenous supply of glucose, the increase of SUMO1/2 accumulation and *SUMO E3 ligase SIZ1* gene expression demonstrated that the SUMOylation might be activated by providing exogenous glucose for the activation of transcription factors. SUMO E3 ligase SIZ1 is accountable for preserving the stability and promoting the transcriptional activity of transcription factors by their SUMOylation and by suppressing their degradation by ubiquitin–proteasome system. Therefore, following the exogenous supplying of glucose or providing endogenous glucose, TOR signaling might be accountable for increasing the activity of SUMO E3 ligase SIZ1, thus suppressing the SnRK1 signaling pathway^8,45^. Therefore, it can be assumed that promoting the glucose-TOR signaling pathway might increase the SUMO1/SUMO E3 ligase SIZ1 signaling pathway in rose flowers during bud opening. Therefore, the greater decorative quality of flowers might be attributed to SUMO1/SUMO E3 ligase SIZ1 signaling pathway achieved from the glucose-TOR signaling pathway. Following harvest, the suppression of the glucose-TOR signaling pathway in cut rose flowers might be accountable for deactivating the SUMO1/SUMO E3 ligase SIZ1 signaling pathway during vase life. Therefore, the lower decorative quality of cut rose flowers during vase life might be attributed to the lower activity of the SUMO1/SUMO E3 ligase SIZ1 signaling pathway that is achieved from the suppression of the glucose-TOR signaling pathway.

In plants, SUMO E3 ligase SIZ1 is accountable for SUMOylation of ICE1 transcription factor, suppressing poly-ubiquitination of ICE1 by E3 ubiquitin ligase HOS1 stabilizing ICE1^4^. In addition, SUMO-ICE1 inhibited MYB15 as a repressor of CBF3/COR expression. SUMOylation of ICE1 by SUMO E3 ligase SIZ1 promotes the CBF3 signaling pathway directly by ICE1 transcriptional activity or indirectly by suppressing MYB15 transcriptional activity^4^. The binding of CBFs to CRT/DRE motif in promoter of *CORs* genes (1) enhances the activity of ROS scavenging systems, (2) ensures a sufficient intracellular supply of ATP, (3) promotes *HSPs* gene expression, and (4) activates the endogenous accumulation of polyamines, proline, and GABA by promoting the activity of arginine pathways^46^. In plants, SUMO E3 ligase SIZ1 dependent SUMOylation enhances the activity of NR enzyme and preserves NR protein stability, which not only improves the activity of glutamine synthetase/glutamate synthase (GS/GOGAT) cycle by supplying NH_4_^+^ but also activates the endogenous accumulation of nitric oxide^10^. SUMO E3 ligase SIZ1 is accountable for SUMOylation of MYB75 and MYB1 transcription factors, which improves their stability for increasing the expression of phenylpropanoid pathway for promoting the accumulation of anthocyanins^13,14^. In addition to anthocyanin accumulation, the supplying of NH_4_^+^ through the activity of phenylalanine ammonia-lyase (PAL) enzyme might be accountable for providing glutamate via GS/GOGAT cycle^47^. In plants, the activity of the NR enzyme is accountable for producing NO_2_^−^ for the activity of nitrite reductase enzyme for supplying NH_4_^+^. In chloroplast or mitochondria, the GS enzyme is accountable for providing glutamine from glutamate and NH_4_^+^. With the activity of the GOGAT enzyme, two glutamate molecules are produced from α-ketoglutarate, and one glutamate molecule is essential for allowing GS/GOGAT cycle to continue. With the activity of the GS/GOGAT cycle, glutamate supply is useful for proline and GABA biosynthesis. In addition, a sufficient intracellular supply of glutamate might be accountable for arginine supplying for starting the activity of the ARG/NOS pathway for the endogenous accumulation of nitric oxide, polyamines, proline, and GABA^48,49^. Therefore, SUMO E3 ligase SIZ1 may increase glutamate supply by promoting the activity of NR/PAL enzymes. The glutamate supply is used by ICE1/CBFs signaling pathway for supporting the biosynthesis proline and GABA. In plants, a sufficient glutamate supply provided by GS/GOGAT cycle might be accountable for proline biosynthesis by the P5CS enzyme. In addition to glutamate, the delivery of ornithine is responsible for proline biosynthesis by the OAT enzyme^48,50^. Endogenous proline serves as an osmoprotectant and exhibits ROS scavenging capacity, which is beneficial for keeping membrane integrity and is also helping as a chaperoning molecule for stabilizing ROS scavenging proteins^48^. In addition to proline, the glutamate supply provided by GS/GOGAT cycle might be accountable for GABA biosynthesis through the activity of the GAD enzyme^51^. In addition to osmoregulation capacity, the endogenous GABA, as an osmoprotectant, exhibits ROS scavenging capacity.

Furthermore, endogenous GABA accumulation by the GAD enzyme prevents cytosolic acidification during senescence and stress^52^. In our study, increasing the activity of ICE1/CBFs transcriptional system accompanied by ensuring a sufficient supply of glutamate through GS/GOGAT cycle by SUMO1/SUMO E3 ligase SIZ1 signaling pathway might be accountable for activating endogenous accumulation of proline and GABA in rose flowers during bud opening, achieving from higher expression and activity of *P5CS* and *GAD*, respectively. Therefore, the superior decorative quality of rose flowers during bud opening might be attributed to the higher endogenous proline and GABA accumulation accompanied by promoting SUMO1/SUMO E3 ligase SIZ1 signaling pathway.

In plants, unfriendly ROS accumulation accelerates the deterioration of membrane integrity by increasing the peroxidation of membrane unsaturated fatty acids as realized by a higher MDA accumulation. For countering oxidative stress, plants have evolved ROS-avoiding and scavenging systems^52^. In plants, SUMOylation as a molecular switch is accountable for maintaining intracellular ROS homeostasis. SUMO E3 ligase SIZ1 is accountable for intracellular ROS homeostasis by increasing expression of alternative oxidase (*AOX*) and uncoupling protein 1 (*UCP1*) genes, which is a ROS avoidance strategy, which also promotes gene expression and expression and activities of ROS scavenging *SOD*, *CAT*, *APX*, and *GR*. In addition, by suppressing endogenous salicylic acid biosynthesis through the isochorismate synthase pathway, SUMO E3 ligase SIZ1 may prevent ROS accumulation by stopping NADPH oxidases^8,11,12^. Mishra et al.^53^ reported that the rice *SUMO E3 ligase SIZ1* gene overexpression conferred heat and drought stress tolerance in cotton plants by improving photosynthesis performance by protecting the activity of the electron transport system, increasing *HSPs* expression, and promoting *SOD* and *APX* expression. Zhang et al.^54^ reported that the tomato *SUMO E3 ligase SIZ1* gene overexpression conferred drought tolerance in tobacco by activating endogenous accumulation of proline achieving from higher *P5CS* gene expression, lower accumulation of O_2_^−^ and H_2_O_2_ achieving from higher expression and activity of *CAT* and *APX*, suppressing chlorophyll degradation and preserving leaf water status, and maintaining membrane integrity as realized by lower MDA accumulation. Zhang et al.^7^ reported that the SUMO E3 ligase SIZ1 gene overexpression conferred heat tolerance while SUMO E3 ligase SIZ1 gene silencing by RNA interference aggregated heat damage in the tomato plants. During heat stress, SIZ1 is accountable for HSFs SUMOylation for increasing *HSP70* and *HSP90* expression. Moreover, SIZ1 might be responsible for stabilizing HSP70 and APX proteins through their SUMOylation. With SUMO E3 ligase SIZ1 gene overexpression, the preservation of membrane integrity as realized by lower MDA accumulation might be attributed to higher endogenous proline accumulation achieved from higher *P5CS* gene expression, higher expression of molecular chaperones *HSP70* and *HSP90* and the accumulation of their proteins, and lower accumulation of O_2_^−^ and H_2_O_2_ achieved from higher *APX* expression and activity^7^. In our study, a sufficient sugar supply in rose flowers during bud opening promotes the glucose-TOR signaling pathway. Then, the activation of SUMO1/SUMO E3 ligase SIZ1 signaling pathway by glucose-TOR signaling pathway employed ICE1/CBFs and HSFs/HSPs transcriptional signaling systems. In plants, ICE1/CBFs and HSFs/HSPs transcriptional activity might be accountable for attenuating endogenous H_2_O_2_ accumulation by promoting ROS scavenging *SOD*, *CAT*, *APX*, and *GR* expression and activity, leading to lower MDA accumulation^55–58^. Therefore, the superior decorative quality of rose flowers during bud opening might be attributed to higher ROS scavenging *SOD*, *CAT*, *APX*, and *GR* expression and activity associated with activating SUMO1/SUMO E3 ligase SIZ1 signaling pathway.

After flowers are harvested, the suppression of the TOR signaling pathway and promoting the SnRK1 signaling pathway by intracellular deficiency of sugar and water stress accelerate petal senescence due to intracellular insufficiency of ATP and NADPH supply and unfriendly intracellular accumulation of ROS. The SnRK1 signaling pathway employs bZIPs transcription factors for orchestrating intracellular sugar and energy homeostasis by intracellular ATP shortage. After flowers are harvested, insufficient sugar supply in rose flowers during petal senescence suppresses the glucose-TOR signaling pathway. By stopping the glucose-TOR signaling pathway, the SnRK1 signaling pathway's increased activity might be accountable for suppressing SUMO1/SUMO E3 ligase SIZ1 signaling pathway in rose flowers during petal senescence. In plants, ICE1/CBFs and HSFs/HSPs transcriptional activity might be accountable for attenuating endogenous H_2_O_2_ accumulation by promoting gene expression and activities of ROS scavenging *SOD*, *CAT*, *APX*, and *GR*. In our study, higher endogenous H_2_O_2_ accumulation arising from lower gene expression and activities of ROS scavenging *SOD*, *CAT*, *APX*, and *GR* in rose flowers during petal senescence was associated with lower *SUMO1* and *SUMO E3 ligase SIZ1* genes expression. As a result, the inferior decorative quality of rose flowers might be attributed to the lower activity of the ROS scavenging system accompanied by the lower SUMO1/SUMO E3 ligase SIZ1 signaling pathway.

In rose flowers during petal senescence, the lower *SUMO1* and *SUMO E3 ligase SIZ1* genes expression was associated with lower *P5CS* expression and activity. Higher *OAT* expression and activity might be responsible for proline accumulation in rose flowers during petal senescence. Kumar et al.^59^ reported that the higher activity of the P5CS enzyme is responsible for endogenous proline accumulation in rose flowers during bud opening. The activity of the GS enzyme is accountable for providing the P5CS enzyme with glutamate. However, lower activity of GS enzyme was concomitant with higher activity of NADH-GDH enzyme, beneficial for the activity of glutamate supplying P5CS and for the detoxification of NH_4_^+^ produced by proteolysis during rose flower senescence. The higher NH_4_^+^ accumulation from the higher proteolysis may serve as a signal for promoting the activity of the NADH-GDH enzyme during rose flower senescence.

Additionally, the higher activity of the OAT enzyme under water deficiency might be accountable for proline biosynthesis from ornithine during rose flower senescence. With the supply of ornithine from proteolysis through the urea cycle during rose flower senescence, supplying one molecule NADPH for pyrroline-5-carboxylate reductase (P5CR) is accountable for proline biosynthesis, whereas providing of one molecule ATP and two molecules NADPH is accountable for proline biosynthesis from glutamate. Therefore, OAT enzyme activity might be responsible for proline biosynthesis from ornithine during rose flower senescence by insufficient intracellular ATP provision accompanied by water deficiency^59^. Therefore, a sufficient endogenous supply of proline by *OAT* expression and activity during petal senescence in rose flowers might be provided by the proline dehydrogenase (ProDH) enzyme for a sufficient intracellular provision of ATP accompanied by friendly ROS accumulation. In plants, activities of ICE1/CBFs and NR/MYBs pathways might be accountable for endogenous proline accumulation by increasing *P5CS* gene expression and enzyme activity along with sufficient intracellular supply of glutamate. In our study, higher endogenous proline accumulation accompanied by lower SUMO1/SUMO E3 ligase SIZ1 signaling pathway in rose flowers during petal senescence might be attributed to higher *OAT* expression and activity. As a result, the inferior decorative quality of rose flowers might be attributed to lower glutamate supply for proline biosynthesis due to lower *P5CS* expression and activity, which was accompanied by lower *SUMO1* and *SUMO E3 ligase SIZ1* genes expression.

In rose flowers during petal senescence, lower *SUMO1* and *SUMO E3 ligase SIZ1* genes expression was associated with lower endogenous GABA accumulation achieved from the higher activity of GABA shunt pathway realized by *GABA-T* and *SSADH* expression and activity. In rose flowers during petal senescence, promoting the activity of GABA shunt pathway might be accountable for allowing the activity of tricarboxylic acid cycle by providing succinate through the activity of SSADH enzyme, leading to sufficient supply of NADH and carbon skeletons. Moreover, the delivery of NADH and succinate by the SSADH enzyme might be accountable for the activity of the mitochondria electron-transporting system, providing a sufficient ATP supply and avoiding ROS accumulation^51,60,61^. In plants, for protein succinylation, the tricarboxylic acid cycle is crucial as it supplies succinate or succinyl-CoA. During rose flower petal senescence, lower activities of α-​ketoglutarate dehydrogenase and succinyl-CoA synthetase enzymes in the tricarboxylic acid cycle led to unfriendly ROS accumulation, which may restrict succinate supply for protein succinylation. Hence, the GABA shunt pathway might be crucial for succinate for succinylation of the tricarboxylic acid cycle and electron transporting system proteins, which is beneficial for supporting a sufficient ATP supply and ROS signaling^62^. With the activity of the GABA shunt pathway, providing sufficient succinate for succinylation is crucial for the glycolysis pathway, tricarboxylic acid cycle, and electron transport system activity for higher ATP supply and lower ROS accumulation which might be beneficial for postponing rose flower petal senescence^63,64^. In plants, ICE1/CBFs and NR/MYBs pathways might be accountable for (1) endogenous accumulation of GABA by increasing *GAD* gene expression and enzyme activity and (2) sufficient intracellular supply of glutamate. In our study, lower endogenous GABA accumulation associated with lower SUMO1/SUMO E3 ligase SIZ1 signaling pathway in rose flowers during petal senescence might be attributed to the higher activity of the GABA shunt pathway by increasing *GABA-T* and *SSADH* expression and activity. As a result, the inferior decorative quality of rose flowers might be attributed to lower glutamate supply for GABA biosynthesis due to lower *GAD* expression and activity, which was accompanied by lower *SUMO1* and *SUMO E3 ligase SIZ1* genes expression. Therefore, a sufficient endogenous GABA supply by *GAD* expression and activity during bud opening in rose flowers might be utilized by *GABA-T* and *SSADH* expression and activity, which is required for intracellular ATP provision and avoiding ROS accumulation in rose flowers during petal senescence.

By serving as a moonlighting protein, the PSKα receptor (PSKR1) exhibits protein kinase and guanylate cyclase activity. Following the administration of PSKα treatment, the cytosolic accumulation of cGMP, the activation of protein kinase G (PKG), and cyclic nucleotide-gated ion channel (CNGC) could be responsible for PSKα signal transduction^65,66^. Aghdam et al.^17^ reported that the PSKα treatment at 150 nM extended the vase life of cut rose flowers due to the cytosolic accumulation of cGMP, which promoted PKG and CNGC1 expression, thus realizing activated PSKα signal transduction for postponing petal senescence. Following the application of 150 nM PSKα, the reinforcement of the endogenous PSKα signaling pathway by suppressing phosphodiesterase (*PDE*) gene expression might be accountable for postponing senescence and extending the vase life of cut rose flowers^1^.

The translocation of HSFs from the cytoplasm to the nucleus is crucial for HSFs binding to heat shock elements (HSEs) in HSPs gene promoters for increasing the expression of HSPs. In addition, HSFs may serve as cellular redox sensors by activating *APX* gene expression for ROS scavenging during senescence^56^. In our study, following the application of 150 nM PSKα treatment, increasing HSFs/HSPs transcriptional activity by SUMO1/SUMO E3 ligase SIZ1 signaling pathway might not only be accountable for increasing the expression of *HSP70* and *HSP90* but might also be accountable for enhancing the activity of ROS scavenging system in cut rose flowers during vase life. *HSPs* expression and protein accumulation serve as intracellular molecular chaperones beneficial for protein synthesis, renaturation, and stabilization. HSPs migration from the cytosol to the membrane during senescence and stress is crucial for keeping membranes integrity and fluidity. In addition, HSPs exhibit ROS scavenging capacity beneficial for keeping membranes fluidity and integrity by protecting fatty acids against peroxidation by ROS accumulation during senescence and stress. In addition, the binding of HSPs to membrane phospholipids and galactolipids is beneficial for keeping membranes fluidity and integrity. By binding to the membrane phospholipids and galactolipids, HSPs preserve membranes integrity by blocking the binding of phospholipase enzymes to membranes. Furthermore, HSPs are crucial for keeping the stability of mitochondrial and chloroplast electron-transporting proteins beneficial for providing a sufficient intracellular supply of ATP and avoiding ROS accumulation. HSPs are useful for intensifying intracellular ROS scavenging capacity by promoting the accumulation of ROS scavenging AA and GSH molecules and the activities of ROS scavenging enzymes^55,56,67,68^. In addition, Gagné et al.^69^ reported that the PARP1 suppresses *HSP70* gene expression by binding to the HSP70 promoter. By heat stress sensing, SUMO E3 ligase SIZ1 and SUMO E4 ligase are accountable for PARP1 polySUMOylation, which targets PARP1 for ubiquitination by ubiquitin E4 ligases for PARP1 degradation by the ubiquitin–proteasome system. With the degradation of PARP1, PARP1 clearance from the HSP70 promoter contributes to *HSP70* gene expression. Therefore, SUMOylation by SUMO E3 ligase SIZ1 might be accountable for regulating the expression of *HSPs*^70^. With the suppression of *PARP1* gene expression following the application of PSKα treatment^21^, the higher activity of the SUMO1/SUMO E3 ligase SIZ1 signaling pathway might be accountable for increasing the expression of *HSP70* and *HSP90* in cut rose flowers during the vase life. Therefore, the superior decorative quality of rose flowers following the 150 nM PSKα treatment application might be attributed to higher expression of *HSP70* and *HSP90* achieved from SUMO1/SUMO E3 ligase SIZ1 signaling pathway. In our study, associated with higher *SUMO1* and *SUMO E3 ligase SIZ1* genes expression, higher expression of *HSP70* and *HSP90* in cut rose flowers during vase life might be accountable for postponing petal senescence.

Developmental factors and hormonal cues such as gibberellin and cytokinin^71–73^, brassinosteroids or ERFs transcriptional activity^74,75^ during bud opening may be responsible for triggering *PSKs* genes expression^1^. By triggering PSKα signaling, promoting PKG, Ca^2+^/CaM or CDPK may be responsible for cytosolic cGMP signal responsive genes expression^1^. Transcription factors stability, DNA binding capacity, and subcellular location orchestration directly by PKG, Ca^2+^/CaM, or CDPK^76^ or indirectly by SUMO1/SIZ1 signaling pathway^77^ may be responsible for ROS scavenging, along with proline, and GABA biosynthesis responsive genes expression. In addition, SUMO1/SIZ1 signaling pathway may serve as a downstream target of PSKα signaling for ROS scavenging, proline, and GABA biosynthesis responsive proteins SUMOylation^4,7–9,12,78–80^ which could support flower decorative quality by maintaining intracellular energy and ROS homeostasis^81–84^. By flower harvesting, developmental factors and hormonal cues such as ABA and ethylene (water shortage stress)^71–73,84^ may be responsible for suppressing PSKα signaling by triggering *PDE* gene expression for preventing cytosolic cGMP signaling or repressing *PSKs* genes expression^1^. By suppressing PSKα signaling, impeding PKG, Ca^2+^/CaM, or CDPK and/or SUMO1/SIZ1 signaling pathway may confine ROS scavenging system activity and proline and GABA biosynthesis. Consequently, disrupting intracellular energy and ROS homeostasis^81,82,84^ may be responsible for decorative quality losing and vase life termination of flowers. Therefore, unfriendly intracellular ROS accumulation and insufficient intracellular ATP supply, probably resulting from confined PKG, Ca^2+^/CaM, or CDPK and/or SUMO1/SIZ1 signaling pathway, are intrinsic features of petals senescence. In addition, reinforcing endogenous PSKα signaling associated with promoting SUMO1/SIZ1 signaling pathway could be responsible for delaying senescence and prolonging the vase life of cut rose flower by exogenous PSKα application. However, deeply research is needed for understanding and elucidating post-translational regulatory mechanisms orchestrating petals senescence in rose flowers.

## Conclusion

**Figure 5:**
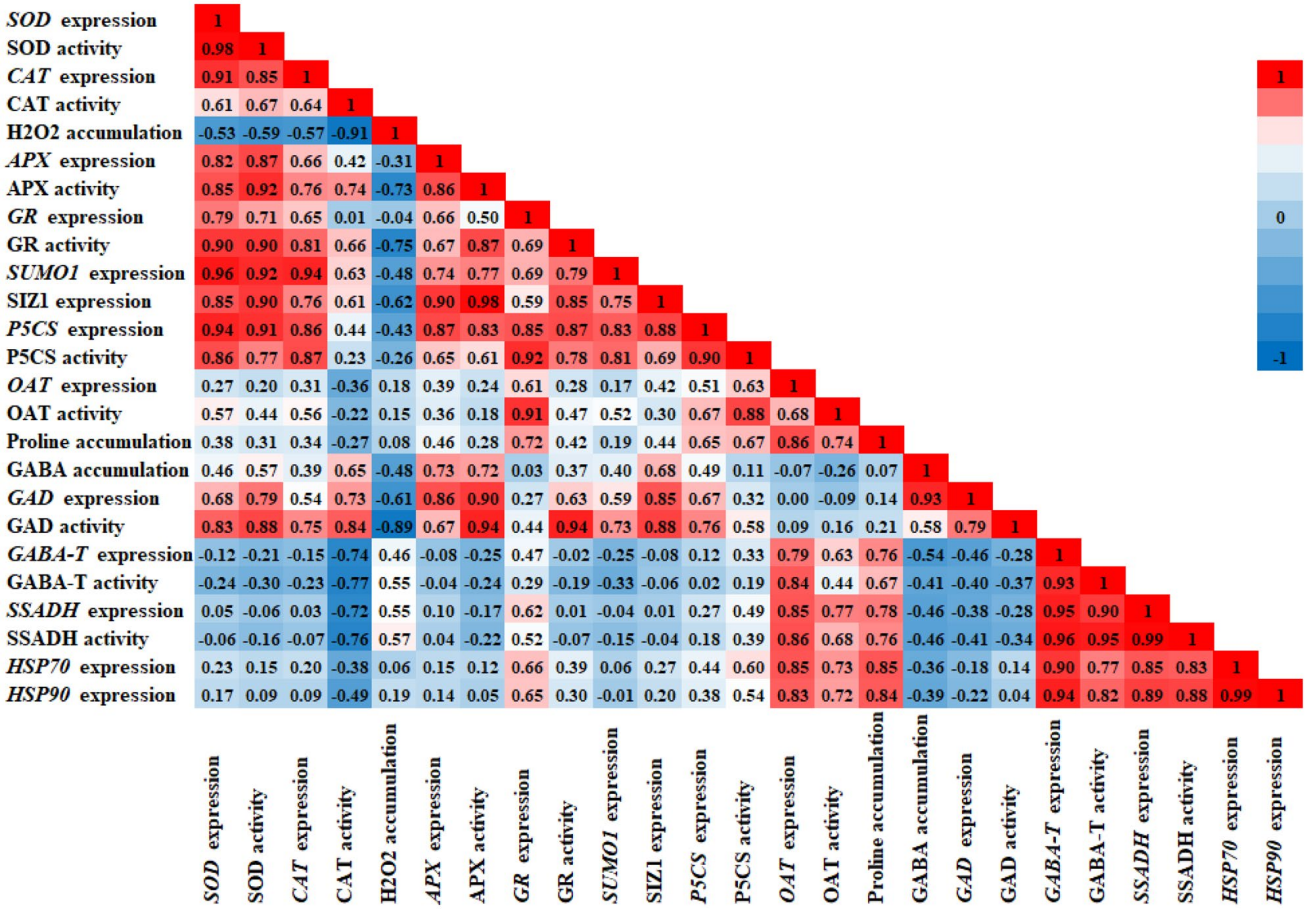
The Pearson correlation coefficient was calculated based on *SUMO1* and *SUMO E3 ligase SIZ1* genes expression, *SOD*, *CAT*, *APX* and *GR* genes expression and enzymes activity, *P5CS* and *OAT* genes expression and enzymes activity, *GAD*, *GABA-T* and *SSADH* genes expression and enzymes activity, and *HSP70* and *HSP90* genes expression accompanying by endogenous H_2_O_2_, proline and GABA accumulation of cut rose flowers in response to 0 and 150 nM PSKα treatment.

In our study, during flower bud opening, higher *SUMO1* and *SUMO E3 ligase SIZ1* genes expression were accompanied by (1) higher expression and activities of ROS scavenging *SOD*, *CAT*, *APX*, and *GR*, (2) higher proline accumulation due to higher *P5CS* gene expression and enzyme activity, and (3) higher GABA accumulation due to higher *GAD* expression and activity. After flowers are harvested, lower activity of SUMO1/SUMO E3 ligase SIZ1 signaling pathway was associated with lower activity of ROS scavenging system and lower glutamate supply for proline and GABA accumulation, thereby accelerating petal senescence in rose flowers. Following the administration of PSKα treatment, postponing petal senescence in cut rose flowers could be ascribed to triggering *SUMO1* and *SUMO E3 ligase SIZ1* genes expression accompanied by higher activity of ROS scavenging system, higher accumulation of proline and GABA, and higher expression of *HSPs* (Fig. 5). Our results highlight the potential of the PSKα to be employed as a promising antisenescence signaling peptide in the floriculture industry to extend the vase life of cut rose flowers.

## Supplementary Information

Supplementary Information.
