# Supplementary material for: Phytosulfokine α (PSKα) delays senescence and reinforces SUMO1/SUMO E3 ligase SIZ1 signaling pathway in cut rose flowers (Rosa hybrida cv. Angelina)
Source: Sci Rep. 2021 Dec 1;11:23227. doi: 10.1038/s41598-021-02712-2 (PMC8636500; doi:10.1038/s41598-021-02712-2)

**Supplementary Fig. 1.** Exogenous PSKα application at 0, 75, 150, 225, and 300 nM and vase life of cut rose flowers at 20 °C. Data shown are mean values of n = 3, and the error bars represent standard errors of the means. Mean values followed by different letters indicate they are significantly different by LSD test at P < 0.05.


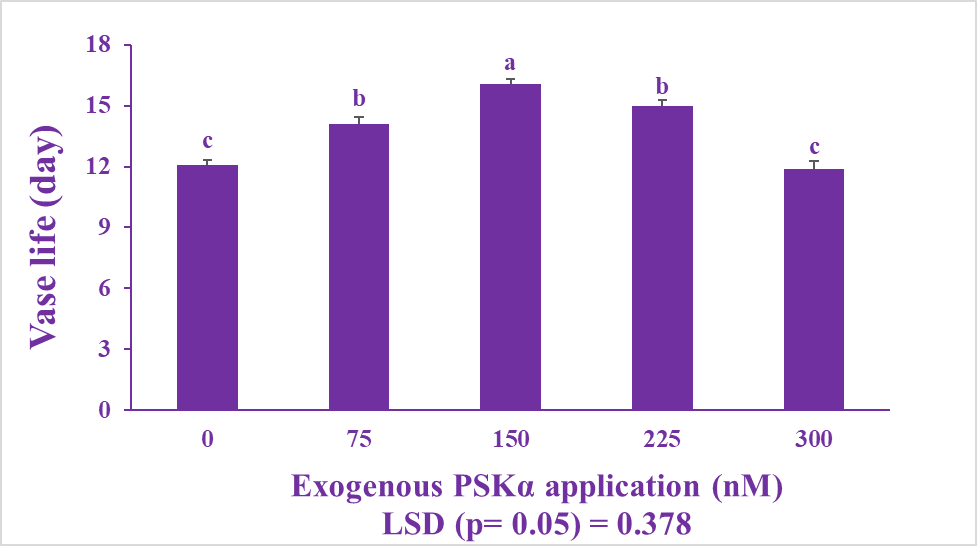

Supplement: Supplementary file 1 — Supplementary Information. [file 41598_2021_2712_MOESM1_ESM.doc]
